# Mechanical Thrombectomy for Large Vessel Occlusion Strokes Involving a Cerebral Aneurysm in the Target Vessel: Case Series

**DOI:** 10.1155/crvm/6073229

**Published:** 2025-04-02

**Authors:** Takeshi Miyazaki, Ryusuke Kori, Masaya Katagiri, Tomoyuki Inoue, Kota Sato, Tatsuya Sato, Yuka Terasawa, Takahiro Himeno

**Affiliations:** ^1^Department of Neurosurgery, Brain Attack Center Ota Memorial Hospital, Fukuyama, Hiroshima, Japan; ^2^Department of Neurology, Brain Attack Center Ota Memorial Hospital, Fukuyama, Hiroshima, Japan

## Abstract

**Objective:** With the increasing prevalence of mechanical thrombectomy (MT) for large vessel occlusion strokes, encountering unruptured cerebral aneurysms (uANs) in MT target vessels has become more common, necessitating case accumulation to establish safety guidelines for MT in such cases. In this study, we aimed to review and present cases of uAN associated with MT target vessels at our hospital.

**Methods:** Among 320 patients who underwent MT for large vessel occlusion strokes at our hospital between January 2018 and December 2021, we selected patients with uAN in the MT target vessel and analyzed various parameters including the occluded vessel, uAN location, timing of uAN discovery, thrombus retrieval procedures, materials, recanalization outcomes, and uAN rupture incidence.

**Results:** Of the 320 patients, 7 had aneurysms in the target vessel (2.2%). The uANs were identified before the device crossed the occluded lesion (lesion crossing (LC)) in four cases, while in three cases, identification occurred after LC or recanalization. In 1 of the 3 cases, a uAN was suspected on preoperative computed tomography at the retrospective review. The thrombectomy procedures included a direct aspiration first pass technique (ADAPT) alone in one patient, stent retrieval (SR) alone in two patients, combination therapy in three patients, and SR combined with local infusion of urokinase in one patient. The effective recanalization rate, defined as TICI 2b or 3, was 57.1% (4/7). The average puncture-to-recanalization time was 77.4 min, and there were no instances of uAN rupture associated with MT.

**Conclusions:** We presented seven cases of uAN in the MT target vessel. No uAN rupture was associated with MT, although the same strategies and techniques of routine MT at our hospital were employed, prioritizing recanalization. Preoperative image assessment considering the possibility of a uAN being present in the MT target vessel is more essential, as well as careful selection of MT procedures according to the situation of each patient.

## 1. Introduction

With the increasing use of mechanical thrombectomy (MT) for large vessel occlusion strokes, the opportunity to encounter uAN in the MT target vessel is becoming more frequent, highlighting the need to ensure the safety of MT in such cases. However, there is a lack of clear guidelines owing to the presence of only sporadic reports on the selection of appropriate procedures. In this study, we aimed to review cases of uAN in MT target vessels at our hospital and share our findings.

## 2. Material and Methods

A retrospective survey was conducted on 320 patients who underwent MT for large vessel occlusion strokes at our hospital between January 2018 and December 2021. Cases were selected based on the presence of a uAN in the target vessel, which was the occluded vessel crossed by the MT device (lesion crossing (LC)). Patients with previously treated uANs were excluded from the study. The investigation focused on the occluded vessel, uAN location, timing of uAN identification, MT procedures, materials employed, recanalization outcomes, and occurrence of uAN rupture in the selected cases. To determine whether the uAN induced rupture, the patient was considered to have no induced rupture if there was no intraoperative extravasation from the uAN and the postoperative CT did not show subarachnoid hemorrhage just around the uAN. Our hospital's policy for MT is to first try a direct aspiration first pass technique (ADAPT) [1] if it is feasible. However, if the ADAPT is unsuccessful or presumed to be unsuitable (the thrombus is expected to extend over a long distance or beyond M2, where the aspiration catheter cannot reach or there is high tortuosity), then a stent is introduced, and the alternative procedure involves an aspiration catheter with the proximal balloon (ASAP) [2] technique. The research within our submission has been approved by the ethics institutional review board of Brain Attack Center Ota Memorial Hospital (Approval No. 278).

## 3. Results

Of the 320 patients who underwent MT, 7 patients (2.2%) had aneurysms in the target vessel (Table 1). None of the seven patients received concomitant intravenous rt-PA. The occluded vessels in these seven cases included the middle cerebral artery (MCA) in two, internal carotid (IC) artery in two, vertebral artery (VA) in one, and basilar artery (BA) in two patients. The uAN sites were observed at the IC–posterior communicating (PC) artery bifurcation in three, MCA bifurcation in one, VA in one, and BA in two cases. In two cases, the uAN was located proximal to the occlusion, and in the other cases, the uAN was located distal to the occlusion. The identification of uAN occurred before LC in four cases (known cases) and after LC or recanalization in three cases (unknown cases). The most dangerous, “distal and unknown” uAn type was in three cases (Cases 2, 5, and 6). One patient underwent ADAPT alone, two underwent stent retrieval (SR) alone, three underwent combination therapy (ASAP), and one underwent SR combined with local urokinase infusion. A J-shaped microwire was never used for LC. The effective recanalization rate for TICI 2b or higher was 4/7 (57.1%). The average puncture-to-recanalization (P-to-R) time was 77.4 min, and no uAN rupture was associated with MT.

### 3.1. Illustrative Case of Known Aneurysm Before LC (Case No. 1)

**Figure 1:**
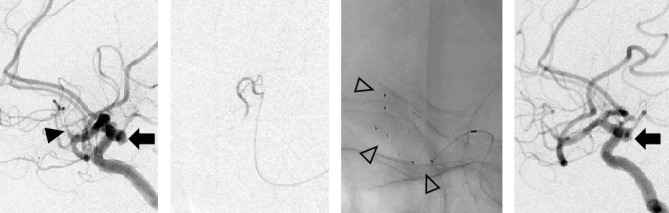
Case 1. (a) Lateral view of left carotid angiography showing simultaneous occlusion of the left MCA (M2 portion) (black arrowhead) and saccular aneurysm at the IC-PC bifurcation (black arrow). (b) Angiography via microcatheter navigated into the distal MCA through the portion of the IC-PC aneurysm. (c) White arrowheads indicating deployed SR. (d) After SR thrombectomy, the saccular aneurysm did not rupture (black arrow). MCA, middle cerebral artery; SR, stent retrieval.

An aneurysm was identified in the proximal part of the occlusion site immediately preceding the angiography (Figure 1). Since the thrombus was distal from M2, SR was selected from the outset instead of ADAPT. The microcatheter was carefully navigated to the distal occlusion site through the IC-PC segment with the advancement of a microwire (Figure 1). Two attempts of SR (Tron) were performed, but effective recanalization was not achieved (Figure 1). The process was completed without rupture (Figure 1).

### 3.2. Illustrative Case of Unknown Aneurysm Before LC (Case No. 5)

**Figure 2:**
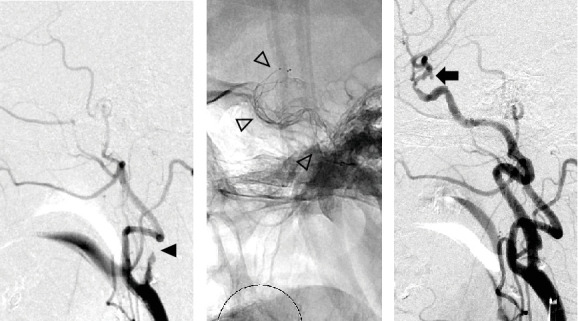
Case 5. (a) Lateral view of left carotid angiography showing occlusion of the left internal carotid artery (black arrowhead). (b) White arrowheads indicating deployed SR. (c) After SR thrombectomy, the saccular aneurysm at the IC-PC bifurcation was visualized (black arrow). SR, stent retrieval.

IC occlusion with a concealed uAN was observed (Figure 2a). Since the occluded distance (thrombus) was estimated to be long, SR was selected from the outset. By advancing a microwire, a microcatheter was navigated to the left IC (C1), and the SR (Trevo 6 mm) was deployed (Figure 2b). Subsequently, a balloon-guiding catheter was introduced into the IC, and blood flow was blocked using a balloon during SR. A large thrombus was successfully retrieved, leading to complete recanalization, which revealed a left IC-PC uAN (Figure 2c).

### 3.3. Illustrative Case of Unknown Aneurysm Before LC (Case No. 6)

**Figure 3:**
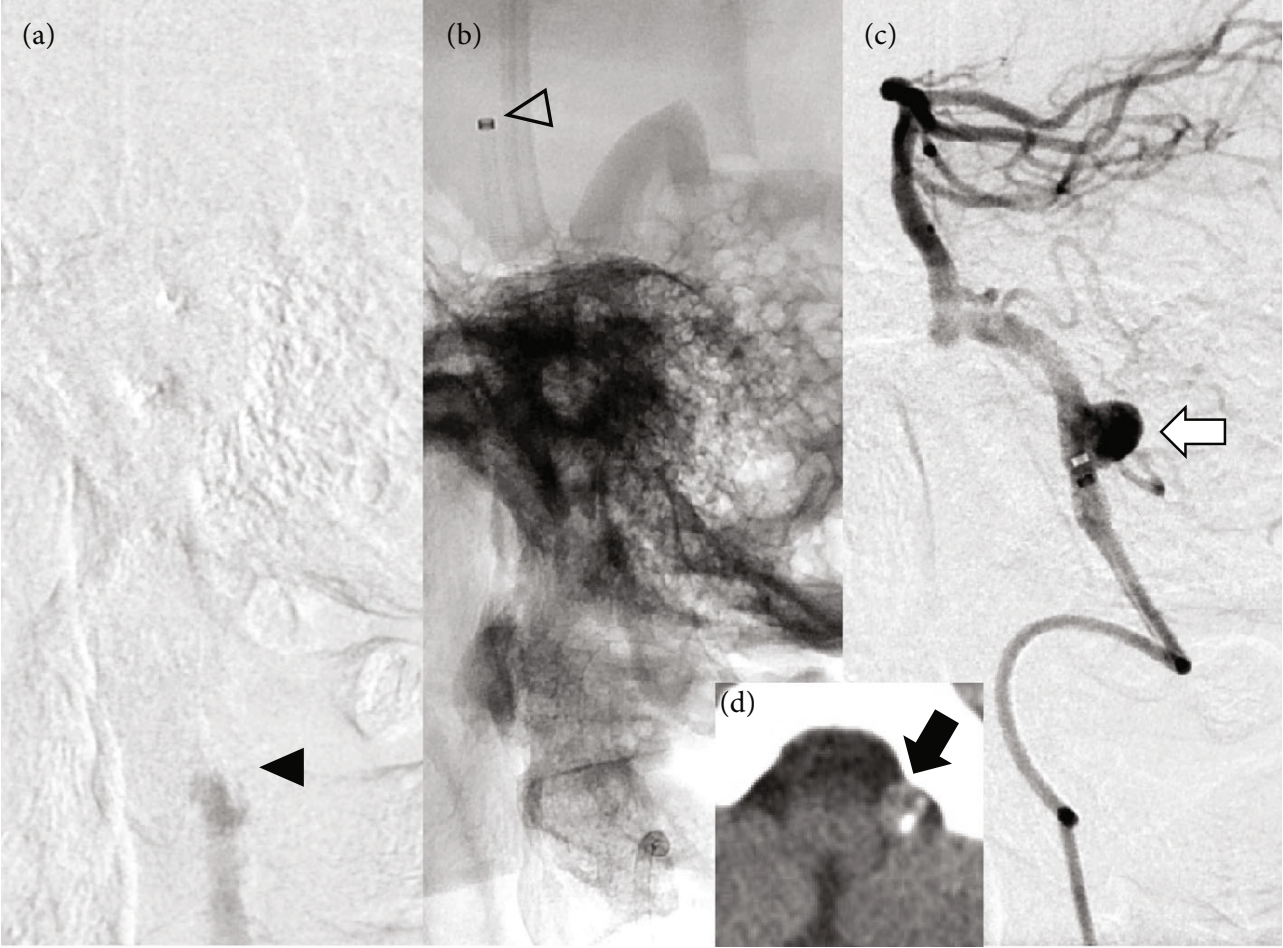
Case 6. (a) Lateral view of left vertebral angiography showing occlusion of the left vertebral artery (black arrowhead). (b) White arrowheads showing the tip of the aspiration catheter navigated to the top of the basilar artery. (c) After aspiration thrombectomy, the saccular aneurysm at the VA-PICA bifurcation was visualized (white arrow). (d) Suspected presence of an aneurysm of the left VA based on preoperative CT findings (black arrow). VA-PICA, vertebral artery-posterior inferior cerebellar artery; VA, vertebral artery; CT, computed tomography.

Vertebrobasilar occlusion with a hidden uAN in the vertebral artery-posterior inferior cerebellar artery (VA-PICA) segment was observed (Figure 3a). Microwires, microcatheters, and aspiration catheters (Catalyst6) were navigated into the occluded left VA. An aspiration catheter was positioned at the top of the BA (Figure 3b). Aspiration thrombectomy (ADAPT) was performed, resulting in complete recanalization. Final angiography revealed the presence of a left VA-PICA uAN (Figure 3c). Retrospectively, suspicious findings of a left VA aneurysm were observed on preoperative CT imaging (Figure 3d).

## 4. Discussion

We reviewed seven patients with uAN in MT target vessels at our hospital. The target vessel and uAN location varied, and ADAPT was used only in one case, while the others were recruited with a stent or stent with an aspiration catheter. The effective recanalization rate was 57.1%. The average P-to-R time was 77.4 min, and no uAN rupture was associated with MT.

### 4.1. Treatment Results in the Cases With uAN in the Target Vessels for MT

In this report, the effective recanalization rate was 57.1%, which was lower than that (82.5%) in all MT cases during the same period (2018–2021) at our hospital (data not shown). The P-to-R time was relatively long, averaging 77.4 min compared with 55.3 min for all MT cases during the same period at our hospital (data not shown). Zibold et al. reported outcomes in 11 patients with uAN in MT target vessels in which effective recanalization was not good (5/11 (45%)) [3]. In contrast, Oshitaka et al. reported seven cases with a good effective recanalization rate of 7/7 (100%), but P-to-R time varied from 27 to 256 min with an average of 76.7 min [4]. In our report, P-to-R time also varied from 15 to 222 min and was more influenced by other potential factors (e.g., access route, length of occlusion, and atherogenicity) rather than delay due to the presence of uAN.

### 4.2. Potential Risk of Inducing Rupture for uAN in the Target Vessels for MT

The rupture risk of uAN in MT procedures involving direct mechanical stimulation of the aneurysm has not been adequately studied despite a few reported cases of rupture [3, 5–7]. Zibold et al. reported the presence of uANs in the MT target vessels in 11 out of 300 MT cases, and rupture occurred in one of these cases, in which an MCA aneurysm rupture occurred, caused by SR [3]. Nozaki et al. also reported one MCA aneurysm case of rupture caused by the SR or microwire among 119 MT cases [5]. Overall, uANs are present in 2%–4% of MT target vessels, and induction of rupture through MT may occur in 0.3%–0.8% of cases. Considering that not all cases of rupture have been reported, this probability is not negligible.

### 4.3. Strategies to Prevent the Rupture of uANs in the MT Target Vessel

**Figure 4:**
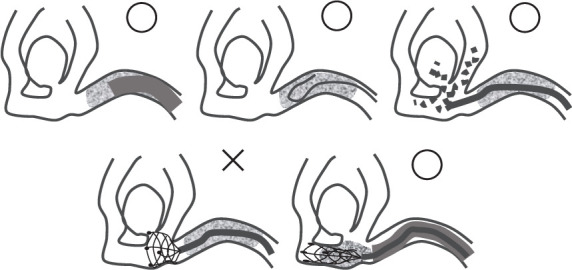
Schematic drawing illustrating strategies to prevent the rupture of uAN on the MT target vessel. (a) ADAPT should be performed first. (b) J-shaped microwire should be used at lesion crossing. (c) Angiography via microcatheter is used to confirm lesion crossing. (d) SR at the aneurysm neck should not be performed. (e) ASAP should be performed to reduce stress on the aneurysm caused by traction force. MT, mechanical thrombectomy; ADAPT, a direct aspiration first pass technique; SR, stent retrieval; ASAP, a stent-retrieving into an aspiration catheter with proximal balloon technique.

Strategies to prevent the rupture of uANs in the MT target vessels have been proposed and reported in the literature. These strategies include the following: (A) performing ADAPT as the first step [4, 8] (Figure 4a), (B) using the J-shaped microwires for the LC [4, 9] (Figure 4b), (C) confirming LC through angiography via a microcatheter [5] (Figure 4c), (D) avoiding SR at the aneurysm neck [4] (Figure 4d), and (E) performing ASAP to reduce stress on the aneurysm caused by traction force [4] (Figure 4e).

Strategies A, B, and C are effective whether or not an aneurysm is known to exist, whereas Strategies D and E should be considered when an aneurysm is known to exist. These proposals appear reasonable, and the appropriate strategy for each case should be selected when possible. However, in real clinical practice, various limitations, such as time constraints and vascular anatomy, often hinder the implementation of these strategies.

As for the shape of the microwire tip during LC, a J-shaped one was not used in any of the seven patients in this study. This is likely because J-shaped microwire tips are not routinely adopted for MT at our hospital. Although the results showed that rupture was not induced even without a J-shaped microwire tip, it may be theoretically better to use a J-shaped microwire tip when an aneurysm is suspected in an MT target vessel. This is because Nozaki et al. reported that rupture might have been induced by microwire perforation [5].

As for MT the procedure, although our hospital's policy contemplates considering ADAPT first, the 7 cases selected for this study included 2 cases of M2 occlusions, which were difficult to reach by aspiration catheter, and 4 cases in which the thrombus was estimated to be long. Several of these cases required stents or concomitant stenting from the beginning. Fortunately, neither of the SR cases, including Case 2 for whom five passages of SR were attempted, resulted in uAN rupture. However, the rupture could have been induced by stress on the neck or by pulling on the adherent thrombus. A potential reason for the absence of rupture in these cases might have been our preference for ASAP use. Other potential reasons include the nature of the two IC-PC aneurysms and one VA aneurysm—which were proximal and unlikely to be displaced by stent traction—and the use of closed-cell stents (Trevo, Solitaire, and Tron), which have a low-risk of catching the stent struts in the neck when deployed across the aneurysm.

### 4.4. Early Detection of uANs in the MT Target Vessels

A uAN existing in the target vessel is classified into distal and proximal types relative to the occluded lesion. In the case of the proximal type, the presence of a uAN could be detected through imaging examinations before MT. In our series, there were two cases of the proximal type (Cases 1 and 3), which were identified on the immediately preceding angiography. The distal type is more dangerous than the proximal type because of the need to cross the occluded lesions, including the uAN, under unpredictable conditions. In our series, there were five cases of the distal type (Cases 2, 4, 5, 6, and 7), but in two cases (Cases 4 and 7), the presence of a uAN could be known using magnetic resonance imaging prior to treatment. Knowledge of the presence of a uAN before MT is crucial for safety, and contrast-enhanced computed tomography angiography [10] and distal imaging from a microcatheter [5] have been advocated. However, in Case 6, whose aneurysm was categorized as “distal” and “unknown,” the presence of a uAN was suspected even with preoperative plain CT from a retrospective perspective. It is important to consider the possibility of a uAN in the target vessel, where MT is to be performed, based on pretreatment imaging studies.

## 5. Conclusion

We presented seven cases of uAN in the MT target vessel. No uAN rupture was associated with MT, although the same strategies and techniques of routine MT at our hospital were employed, prioritizing recanalization. Preoperative image assessment considering the possibility of uAN presence in the MT target vessel is essential, as well as careful selection of MT procedures according to the situation of each patient.

## Figures and Tables

**Table 1:** Clinical and mechanical thrombectomy data of patients involving a cerebral aneurysm in the target vessel.

**Case no.**	**Age (sex)**	**IV rt-PA**	**Occlusion site**	**Size of aneurysm (mm)**	**Aneurysm location**	**Location of aneurysm relative to occluded lesion**	**Known or unknown uAN before LC**	**MT procedure**	**Used microcatheter and guidewire**	**Employing J-shaped microwire**	**Number of passes across aneurysm**	**P to R (min)**	**TICI grade**	**Rupture**
1	71 (F)	No	Lt MCA(M2)	4.4	Lt IC-PC	Proximal	Known by preceding AG for MT	SR (Tron)	Trevo PRO 14 CHIKAI 315 EXC	No	2	35	1	No
2	80 (M)	No	Lt IC	4.2	Lt IC-PC	Distal	Unknown	ASAP (Solitaire)	Rebar18 Transcend EX	No	5	100	3	No
3	69 (F)	No	Rt MCA(M2)	3.2	Rt MCA	Proximal	Known by preceding AG for MT	SR(ReVive) + UK-ia	Prowler Select Plus Transcend EX floppy	No	2	222	2b	No
4	77 (F)	No	BA	2.6	BA-top	Distal	Known by MRI before onset	ASAP (Solitaire)	Velocity Transcend EX	No	1	109	2b	No
5	70 (F)	No	Rt IC	2.3	Rt IC-PC	Distal	Unknown	SR (Trevo)	Velocity Transcend EX	No	1	15	3	No
6	60 (M)	No	Lt VA	7.5	Lt VA-PICA	Distal	Unknown	ADAPT	Velocity Transcend EX	No	1	21	3	No
7	80 (M)	No	BA	15.4	BA fusiform	Distal	Known by MRI at onset	ASAP (Solitaire)	Velocity CHIKAI black/14 soft tip	No	2	40	2a	No

Abbreviations: ADAPT, a direct aspiration first pass technique; AG, angiography; ASAP, a stent-retrieving into an aspiration catheter with proximal balloon technique; LC, lesion crossing; MT, mechanical thrombectomy; P to R, puncture to recanalization; SR, stent retrieval; TICI, thrombolysis in cerebral infarction grade.

## Data Availability

The patient's clinical data used to support the findings of this study are included within the article.
